# An aberrant nuclear localization of E-cadherin is a potent inhibitor of Wnt/β-catenin-elicited promotion of the cancer stem cell phenotype

**DOI:** 10.1038/oncsis.2015.17

**Published:** 2015-06-15

**Authors:** Y-J Su, Y-W Chang, W-H Lin, C-L Liang, J-L Lee

**Affiliations:** 1 Institute of Molecular and Cellular Biology, National Tsing Hua University, Hsinchu, Taiwan; 2 Department of Orthopedics, National Taiwan University Hospital Hsin-Chu Branch, Hsinchu, Taiwan; 3 Department of Medical Science, National Tsing Hua University, Hsinchu, Taiwan

## Abstract

Several studies suggest that Wnt signaling contributes to reprogramming and maintenance of cancer stem cell (CSC) states activated by loss of membranous E-cadherin expression. However, E-cadherin's exact role in Wnt/β-catenin-mediated promotion of the CSC phenotype remains unclear. Recently, a significant positive correlation has been observed between the expression of nuclear (an aberrant nuclear localization) E-cadherin and β-catenin in gastric and colorectal carcinomas. Here we conducted a series of *in-vitro* and *in-vivo* studies to show that the β-catenin/TCF4 interaction was abolished by E-cadherin and was correlated with its nuclear localization, and consequently decreased β-catenin/TCF4 transcriptional activity. Nuclear E-cadherin was a negative regulator of Wnt/β-Catenin-elicited promotion of the CSC phenotype. Using immunohistochemistry on lung cancer tissue microarrays, we found that changes in subcellular location of E-cadherin may be described by tumor grade and stage, suggesting cellular redistribution during lung tumorigenesis. Furthermore, nuclear E-cadherin expression was more significantly inversely correlated with CD133 (a lung CSC marker) expression (*P*<0.005) than total E-cadherin expression (*P*<0.05), suggesting that lung cancer as defined by nuclear E-cadherin^Low^/nuclear β-catenin^High^/CD133^High^ biomarkers has superior prognostic value over total E-cadherin^Low^/nuclear β-catenin^High^/CD133^High^.

## Introduction

Cancer stem cells (CSCs), also called tumor-initiating cells, refer to a small minority of neoplastic cells within a tumor. CSCs are defined by their ability to self-renew, form tumor spheres and seed new tumors in a xenotransplant system, and they are intrinsically resistant to therapy.^1, 2^ The first evidence for the existence of CSCs came from studies on acute myeloid leukemia.^3, 4^ Subsequent studies demonstrated CSCs are also present in solid tumors.^5, 6, 7, 8^ The CSC hypothesis is consistent with clinical data, as current therapies probably kill the majority of tumor cells but leave CSCs unaffected, thus leaving a reservoir of cells that may promote tumor recurrence.^9, 10^ Therefore, elucidation of the detailed molecular mechanisms that regulate CSC formation may lead to more efficacious therapeutic targets.

The Wnt/β-catenin signaling pathway is crucial for the maintenance and activation of normal stem cells, as clearly illustrated with the intestinal mucosa epithelia, epidermis, mammary gland^11, 12^ and other tissues.^13^ Incidentally, important evidence of Wnt signaling has been emphasized in cancer cells^14^ and the Wnt/β-catenin signaling cascade is a critical regulator not only of normal stem cells but also of CSCs.^15^ In non-small cell lung cancer, β-catenin and adenomatous polyposis coli (APC) mutations are uncommon, but Wnt signaling is important in non-small cell lung cancer cell lines, and Wnt inhibition reduces tumorigenic potential.^16^ Roles for Wnt signaling have been described in a variety of CSC settings, including colon, breast and cutaneous CSCs, as well as in hematopoietic stem cells.^15, 17, 18, 19^ For example, stem-like colon cells with a high level of β-catenin signaling have a much greater tumorigenic potential than counterpart cells with low β-catenin signaling.^17^ More specifically, a recent study provides evidence that prostate cancer cells may be regulated by Wnt activity to enhance self-renewal capacity with stem cell characteristics.^20^

Loss of E-cadherin expression often results in the disruption of cell–cell adhesion and an increase in nuclear accumulation of β-catenin. The latter is kept at a low level through a destruction complex;^15^ thus, it may be correlated with tumor grade and stage.^21, 22^ Some evidence shows that the nuclear accumulation of β-catenin is correlated with the expression of CD44 the CSCs markers.^23^ This indicates that activation of β-catenin is required for the sustention of CSC-like traits generated by loss of E-cadherin expression first. E-cadherin can be downregulated through a variety of mechanisms including mutation, epigenetic silencing and transcriptional repression. E-cadherin-inactivating mutations were first described in diffuse gastric cancer^24^ and loss of the E-cadherin locus on the long arm of chromosome 16 (16q22) occurs frequently in hepatocellular carcinomas, breast and esophageal cancer.^25^ However, in colorectal cancer E-cadherin does not always show absent or reduced expression, but often there is redistribution from cell membrane to the cytoplasm.^26^ Failure to localize to the membrane in spite of normal expression may be due to posttranscriptional modification of the complex.^27, 28^ Mutations or loss of heterozygosity within the E-cadherin gene are rare in sporadic colorectal cancer (CRC).^29^ A panel of 49 human colorectal carcinoma cell lines has been screened and it was found that only 7% exhibited inactivating E-cadherin mutation.^30^

Together, this evidence suggests a possible link between cancer stem-like properties and loss of E-cadherin expression. The graded loss expression of E-cadherin correlates with the aggressiveness of numerous carcinomas and the worsening of prognosis, whereas ectopic expression of E-cadherin suppresses tumor initiation and invasion in various *in-vitro* and *in-vivo* tumor model systems. Although this is the first report of nuclear E-cadherin in human lung cancer, aberrant staining of E-cadherin in the nucleus has been reported in other types of cancer including Merkel cell carcinoma, signet ring cell carcinoma and solid pseudopapillary tumors of the pancreas.^31, 32, 33^ Furthermore, those cancers with nuclear E-cadherin also show increased β-catenin levels in the nucleus.^31, 32, 33^ It is unclear whether an aberrant nuclear localization of E-cadherin has an anti- or pro-oncogenic role, for example, via modulating β-catenin-mediated signaling. In this study, we attempted to dissect the molecular mechanisms through which nuclear E-cadherin may have an important role in generating cells with CSC properties. We found that the β-catenin/TCF4 interaction was abolished by E-cadherin and correlated with its nuclear localization, and consequently decreased β-catenin/TCF4 transcriptional activity. Nuclear E-cadherin was a negative regulator of Wnt/β-catenin-elicited promotion of the CSC phenotype. In clinical use, nuclear E-cadherin^Low^/nuclear β-catenin^High^/CD133^High^ biomarkers have superior prognostic value over total E-cadherin^Low^/nuclear β-catenin^High^/CD133^High^.

## Results

### Lung cancer defined by nuclear E-cadherin^Low^/nuclear β-catenin^High^/CD133^High^ biomarkers has superior prognostic value over total E-cadherin^Low^/nuclear β-catenin^High^/CD133^High^

**Figure 1:**
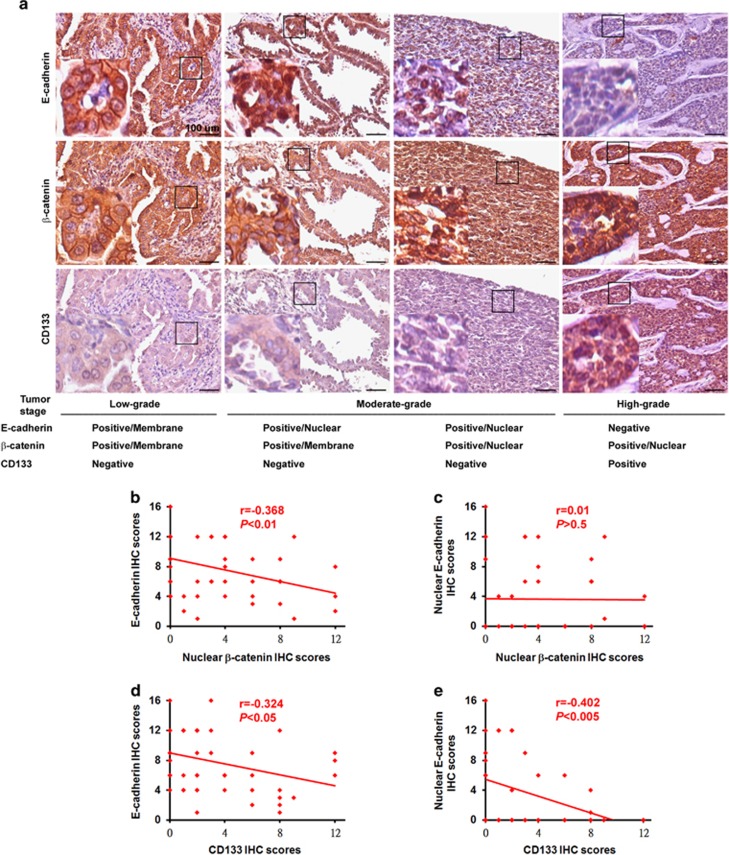
Clinical significance of E-cadherin, β-catenin and CD133 in lung cancer patients. (**a**) Immunohistochemistry (IHC) for E-cadherin, β-catenin and CD133 in representative tumor tissues among 39 human primary and 10 metastatic lung cancer specimens. Both membranous and nuclear positivity of E-cadherin and β-catenin were considered significant. Staging of the primary cancers was carried out according to the AJCC Cancer Staging Manual (7th Edition). Tissues were counterstained in hematoxylin. Scale bars=100 μm. Left bottom panels showed enlarged images from boxed areas as indicated. (**b–e**) Correlations of IHC intensity scores of various proteins. IHC scores=% of positive cells × staining intensity. The graphs were drawn based on the scores presented. Correlations among variables were evaluated using Spearman's rank correlation.

To validate the clinical relevance of E-cadherin and the Wnt/β-catenin pathway to human cancer and its contribution to promoting the CSC phenotype during lung tumorigenesis, we analyzed the expression profiles of E-cadherin, β-catenin and CD133 by immunohistochemical (IHC) staining and scoring;^34, 35^ consecutive slides were examined from 39 primary (stage IA, IB, IIA, IIB, III and IV) and 10 metastatic human lung cancer specimens. Low-grade (stage IA and IB) primary lung cancers showed low levels of nuclear E-cadherin, nuclear β-catenin and CD133, and high levels of membranous E-cadherin and membranous β-catenin. Alternately, moderate-grade (stage IIA and IIB) primary lung cancers showed low levels of CD133 and low-to-moderate levels of nuclear β-catenin, and high-to-moderate levels of nuclear E-cadherin (Figure 1a). In contrast, high-grade (stage III and IV) primary and metastatic lung cancers showed high levels of nuclear β-catenin and CD133, and low levels of total E-cadherin. IHC scoring was determined by multiplying the staining intensity by the percentage of positive tumor cells.^34, 35^ In addition, we found that E-cadherin (total) expression in lung cancers correlated inversely with nuclear β-catenin expression (*P*<0.01; Figure 1b), whereas no correlation was observed between the expression of nuclear E-cadherin and nuclear β-catenin (Figure 1c). Interestingly, nuclear E-cadherin expression was more significantly inversely correlated with CD133 expression (*P*<0.005; Figure 1e) than E-cadherin (total) expression (*P*<0.05; Figure 1d). Patients displaying the signature expression pattern of total E-cadherin^Low^/nuclear β-catenin^High^/CD133^High^ had a survival advantage (*P*=0.004; Supplementary Figure 1a). As expected, patients displaying the signature expression pattern of nuclear E-cadherin^Low^/nuclear β-catenin^High^/CD133^High^ more significantly predicted overall survival intervals (*P*<0.001; Supplementary Figure 1b), suggesting that lung cancer defined by nuclear E-cadherin^Low^/nuclear β-catenin^High^/CD133^High^ biomarkers has superior prognostic value than total E-cadherin^Low^/nuclear β-catenin^High^/CD133^High^.

### Wnt signaling can promote the CSC phenotype in various types of cancer cell lines

**Figure 2:**
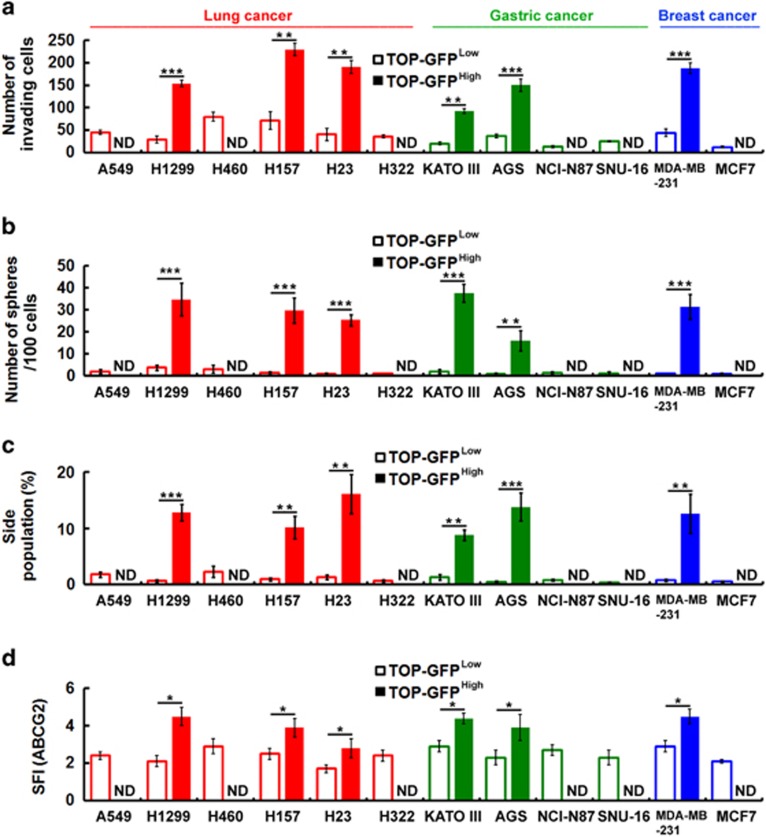
Wnt signaling can promote the CSC phenotype in various types of cancer cell lines. (**a–d**) The highest and lowest 10% of TOP-GFP-expressing cell fractions were isolated by flow cytometry of TOP-GFP-transduced cell lines after treatment with Wnt3a-containing medium. Transwell invasion assays were performed to assess the invasive activity (**a**). The number of spheres formed was quantified (**b**). Cells were stained with Hoechst 33342. SP cells were counted (**c**). Cell surface expression of ATP-binding cassette (ABC)G2 was assessed by flow cytometry using anti-ABCG2 antibody (**d**). The specific fluorescence index (SFI) was calculated as the ratio of the geometric mean fluorescence value obtained with the specific antibody and the isotype control antibody. ND, not detectable. Data were derived from three independent experiments and are presented as the mean±s.d. *P<0.05; **P<0.01; ***P<0.005 (*t*-test).

To investigate the relationship between Wnt/β-catenin signaling and the CSC phenotype, we used a lentivirus-based reporter system for TCF-dependent transcription in which wild-type (TOP) or mutant (FOP) TCF-binding sites were used to drive the expression of enhanced green fluorescent protein. Next, we isolated the highest and lowest 10% of TOP-GFP-expressing cell fractions by flow cytometry of TOP-GFP-transduced cell lines with (Supplementary Figure 2, bottom panel) or without (Supplementary Figure 2, top panel) Wnt3a treatment. To characterize the effects of Wnt signaling with respect to inducing CSC properties, we first examined whether it was capable of enhancing invasiveness, sphere-forming abilities, side population (SP) cells and expression of ABCG2 (the ATP-binding cassette (ABC) transporter family). In the TOP-GFP^High^ fraction, the invasion abilities (Figure 2a and Supplementary Figure 3), the sphere-forming ability (Figure 2b), the SP percentage (Figure 2c and Supplementary Figure 4) and the expression level of ABCG2 (Figure 2d and Supplementary Figure 5) were much higher than those of the TOP-GFP^Low^ fraction in many cancer cell lines. Similar results were shown previously in colon cancer cell lines (Supplementary Figure 6).

### E-cadherin is a negative regulator of Wnt/β-catenin-elicited promotion of the CSC phenotype

**Figure 3:**
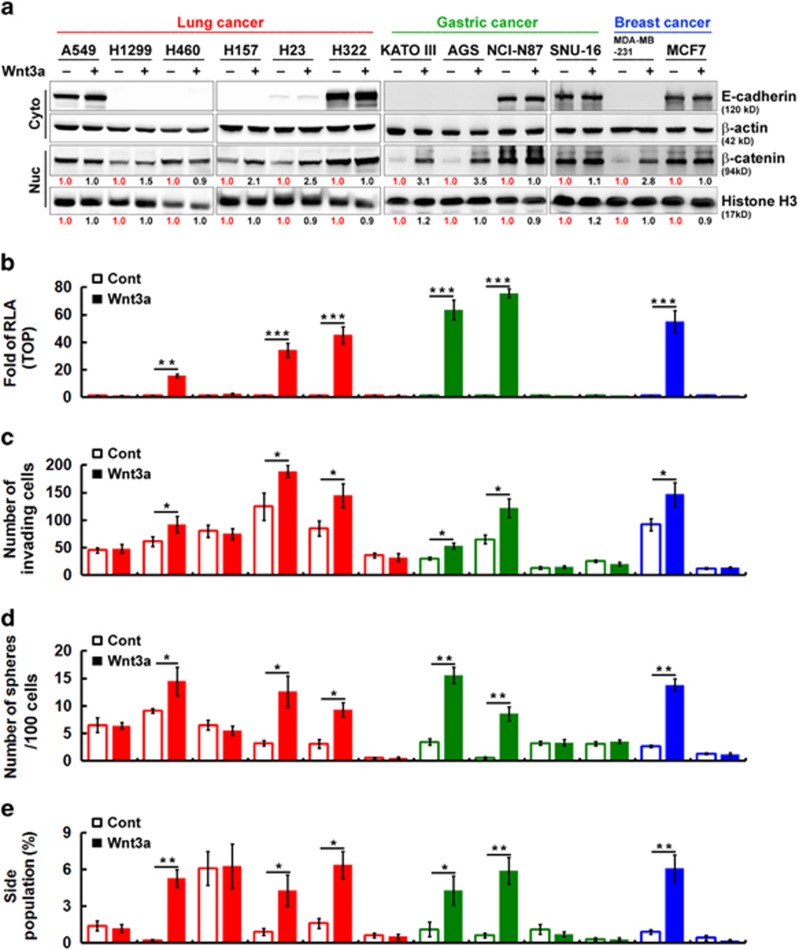
E-cadherin is a negative regulator of Wnt/β-catenin-elicited promotion of the CSC phenotype. (**a**) Western blotting analysis of nuclear (Nuc) and cytosolic (Cyto) fractions of human cancer cell lines after treatment with control or Wnt3a-containing medium. The relative intensities of the bands are shown. (**b**) TOPflash luciferase reporter assays were performed in cells after treatment with control (Cont) or Wnt3a-containing medium. RLA, relative luciferase activity. (**c**) Transwell invasion assays were performed to assess the invasive activity in cells after treatment with control or Wnt3a-containing medium for 20 h. (**d**) The number of spheres formed was quantified in cells after treatment with control or Wnt3a-containing medium. (**e**) After treatment with control or Wnt3a-containing medium, cells were stained with Hoechst 33342. SP cells were counted. Data in **b**–**e** were derived from three independent experiments and are presented as the mean±s.d. *P<0.05; **P<0.01; ***P<0.005 (*t*-test).

As shown in Figure 2, in the TOP-GFP^High^ fraction the properties of CSCs were much higher than those of the TOP-GFP^Low^ fraction in cancer cell lines that seemed to express low E-cadherin levels, whereas differences were not observed in cancer cell lines that expressed high E-cadherin levels. In addition, western blotting demonstrated that nuclear β-catenin was significantly increased only in cancer cell lines that expressed low levels of E-cadherin after stimulation with Wnt3a as compared with cells treated with control medium (Figure 3a). Next, we conducted a luciferase reporter assay (using TOPflash luciferase reporter plasmids) to measure Wnt/β-catenin-mediated transcriptional activation. We detected a low level of β-catenin/TCF-mediated activation in all cancer cell lines maintained in control medium (Figure 3b). Adding Wnt3a into cancer cell lines with low E-cadherin expression significantly increased TOPflash reporter activity; this was consistent with previous data showing that nuclear β-catenin expression is responsive to Wnt stimulation. To characterize the effects of E-cadherin with respect to Wnt signaling-induced CSC properties, we first examined whether it can affect invasiveness, sphere-forming abilities and SP cells. We speculated whether E-cadherin was a negative regulator of Wnt/β-catenin-elicited promotion of the CSC phenotype. Interestingly, we found that cells had distinct invasive abilities (Figure 3c). However, only cells expressing low E-cadherin showed greater invasive abilities after stimulation with Wnt3a (Figure 3c). In addition, we found that the proportion of sphere-forming cells with low E-cadherin expression significantly increased after stimulation with Wnt3a (Figure 3d). As shown in Figure 3e, only cells with low E-cadherin expression exhibited significantly increased SP cells after stimulation with Wnt3a. These findings indicate that E-cadherin may act as a negative regulator in Wnt/β-catenin signaling-elicited promotion of the CSC phenotype.

### Functional fractionation of A549 lung cancer cells by invasive assays

**Figure 4:**
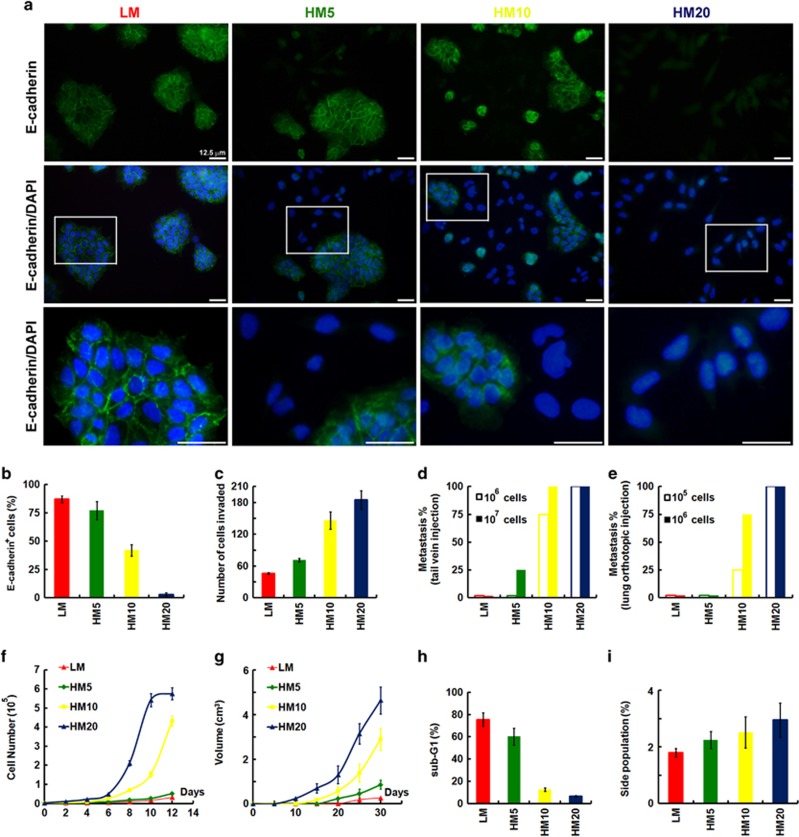
Functional fractionation of A549 lung cancer cells by invasive assays. (**a**) Confocal laser microscopic analysis was performed on A549 cells cultivated in suspension for 24 days and then migrated back onto the plate to reform a monolayer (LM cells). LM cells were functionally fractionated by invasive assays, 5 times (HM5 cells), 10 times (HM10 cells) and 20 times (HM20 cells). Bottom panel showed enlarged images from boxed areas as indicated. Scale bars=25 μm. Immunostaining for E-cadherin is shown in cells; nuclei were counterstained with DAPI (4',6-diamidino-2-phenylindole; lower panels). (**b**) Cell surface expression of E-cadherin was assessed by flow cytometry using anti-E-cadherin antibody in cells described in **a**. (**c**) Transwell invasion assays were performed on cells described in **a**, to assess the invasive activity. For experimental metastasis assays, cells described in **a** in 100 μl phosphate-buffered saline (PBS) were injected into the tail vein (**d**) or the right lung lobes (**e**) of mice. Mice were killed 6–8 weeks after injection and the left lung lobes were embedded in paraffin wax. The proliferation rate of cells described in **a** was determined *in vitro* (**f**) and *in vivo* (**g**). (**h**) Cells described in **a** were cultured in suspension for 120 h before apoptosis assays by flow cytometric analyses of sub-G1 fractions. (**i**) Cells described in **a** were stained with Hoechst 33342. SP cells, excluding Hoechst 33342, were determined. Data in **b**, **c** and **f**–**i** were derived from three independent experiments and presented as means±s.d.

Although the A549 cancer cell line was initially established from a single-cell clone, it has probably become heterogeneous after long-term culture *in vitro*, owing to the genetic instability of cancer cells. Thus, it is possible to isolate populations that have different characteristics from this cell line, in particular the CSC population. A recent report suggests that there may be a direct link between E-cadherin and the acquisition of stem cell properties. In addition, the ability of a subset of cells to become locally invasive suggests loss of cadherin-mediated cell–cell adhesion within the tumor. To enrich the E-cadherin-deficient population by functional fractionation, A549 cells were cultivated in suspension for 24 days and then migrated back onto the plate to reform a monolayer (LM cells). LM cells were functionally fractionated by invasive assays, 5 times (HM5 cells), 10 times (HM10 cells) and 20 times (HM20 cells). Figure 4a shows the morphologic changes in cells as seen under a confocal laser microscope after invasion selection. The parental LM cells had typical epithelial-like cell morphology. However, after serial selection the cells were seen to be larger and exhibited a more mesenchymal spindle shape in culture as compared with LM cells (Figure 4a). Most importantly, cells after serial selection dramatically decreased their expression of E-cadherin (Figures 4a and b). To further determine the phenotype and behavior of the cells after functional fractionation, *in-vitro* invasive, *in-vivo* metastatic abilities, *in-vitro* proliferative potential, *in-vivo* tumor growth, apoptotic abilities and SP cells were determined. After serial selection, cells revealed increased invasive abilities (Figure 4c). For *in-vivo* experimental metastasis assays, cells in 100 μl phosphate-buffered saline were injected into the tail vein (Figure 4d) or the right lung lobes of mice (Figure 4e). The mice were killed 6–8 weeks after injection and the left lung lobes were embedded in paraffin wax. Consistent with previous data, HM20 cells revealed significantly increased metastatic abilities (Figures 4d and e). As cells after serial selection underwent a stable morphological transition that may reflect reprogramming, we performed cell proliferation assays to examine the proliferative ability and behavior of the cells, and compared these results with low invasive cells (LM cells). Surprisingly, the cells after selection exhibited increased proliferation *in vitro* (Figure 4f) and *in vivo* (Figure 4g). In addition, HM20 cells possessed stronger survival signaling resistant to anoikis (Figure 4h). Interestingly, HM20 cells only slightly increased SP as compared with LM cells (Figure 4i), suggesting that loss of E-cadherin expression is not sufficient to generate cells with CSC properties, and that regulation of the CSC phenotype requires additional elements.

### Wnt signaling can be reactivated and promote the CSC phenotype only in E-cadherin-deficient cells

**Figure 5:**
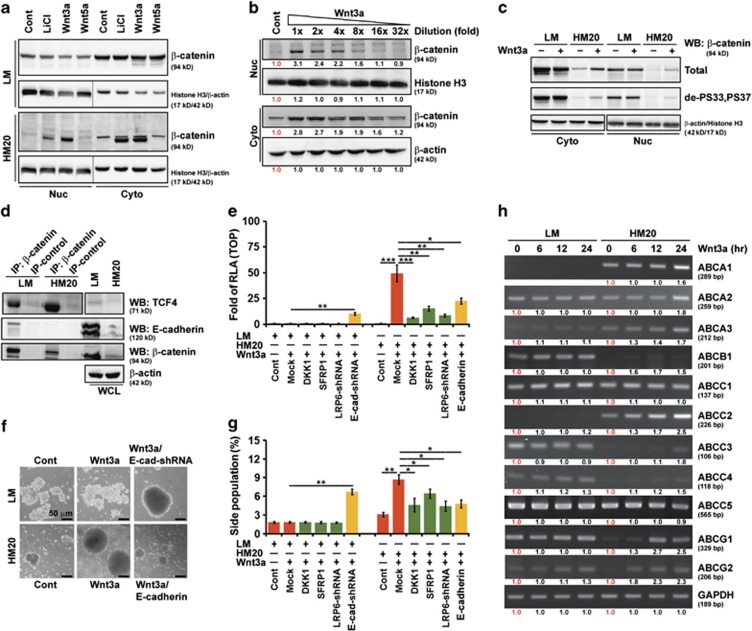
Wnt signaling can be reactivated and promote the CSC phenotype only in E-cadherin-deficient cells. A549 cells were cultivated in suspension for 24 days and then migrated back onto the plate to reform a monolayer (LM cells). LM cells were functionally fractionated by invasive assays, 5 times (HM5 cells), 10 times (HM10 cells) and 20 times (HM20 cells). (**a**) Western blotting (WB) analysis of nuclear (Nuc) and cytosolic (Cyto) fractions of LM and HM20 cells after treatment with control, LiCl, Wnt5a or Wnt3a-containing medium. (**b**) WB analysis of Nuc and Cyto fractions of HM20 cells after treatment with decreasing concentrations of Wnt3a. The relative intensities of the bands are shown. (**c**) WB analysis of Nuc and Cyto fractions of LM and HM20 cells after treatment with control or Wnt3a-containing medium. de-PS33,PS37: dephosphorylated-S33,S37 β-catenin; PS33,PS37,PT41: phosphorylated-S33,S37,T41 β-catenin. (**d**) Whole-cell lysates (WCLs) were prepared from LM and HM20 cells and then IP using anti-β-catenin was carried out followed by WB analysis. (**e**) WCLs were prepared from LM or HM20 cells infected with a lentivirus encoding an shRNA targeting *LRP6* and *CDH1*, or transfected with plasmids encoding DKK1, SFRP1 and E-cadherin, before Wnt3a treatment. A TOPflash luciferase reporter assay was performed. RLA, relative luciferase activity. (**f**) Microscopic analysis of spheres cultivated in suspension for 12 days; spheres were derived from LM and HM20 cells after treatment with control or Wnt3a-containing medium. LM or HM20 cells infected with a lentivirus encoding an shRNA targeting *CDH1*, or transfected with plasmids encoding E-cadherin, before Wnt3a treatment. (**g**) Cells described in **e** were stained with Hoechst 33342. SP cells were counted. (**h**) mRNA was prepared from LM and HM20 cells in the presence of Wnt3a for the indicated times. Expression of the ATP-binding cassette (ABC) transporter family genes was evaluated by reverse transcriptase–PCR. The relative intensities of the bands are shown. Data in **e** and **g** were derived from three independent experiments and are presented as the mean±s.d. *P<0.05; **P<0.01; ***P<0.005 (*t*-test).

To determine whether Wnt/β-catenin signaling could be reactivated in LM (E-cadherin proficient) and HM20 (E-cadherin deficient) cells, the expression of nuclear β-catenin was determined after stimulation with LiCl, Wnt3a, or Wnt5a for 4 h. Western blotting demonstrated that nuclear β-catenin was consistently and strongly overexpressed in HM20 cells after stimulation with Wnt3a and LiCl as compared with cells treated with control medium or Wnt5a (Figure 5a). Further, serial dilutions of Wnt3a treatment in HM20 cells indicated that a one- or twofold dilution was optimal for Wnt/β-catenin signaling reactivation (Figure 5b).

Degradation of β-catenin is dependent on its phosphorylation by GSK3β and CK1α while bound to axin.^36^ We therefore tested whether Wnt3a treatment enhanced the dephosphorylation of β-catenin. The amount of active β-catenin (dephosphorylated at Ser33 and Ser37) was increased by Wnt3a treatment in HM20 but not in LM cells (Figure 5c). It has been reported that β-catenin associates with TCF.^37^ We checked in our cell systems whether β-catenin associated with TCF4 or other proteins. Co-immunoprecipitation (IP) experiments carried out in LM and HM20 cells showed that β-catenin did indeed associate with TCF4 and with E-cadherin as well (Figure 5d). In addition, association of β-catenin with TCF4 inversely correlated with E-cadherin expression, because TCF4 was significantly decreased in β-catenin immunoprecipitates from LM cells, which had very high levels of E-cadherin (Figure 5d).

Next, adding Wnt3a into low E-cadherin-expression (HM20) cells significantly increased TOPflash reporter activity, consistent with our data showing that nuclear β-catenin expression was responsive to Wnt3a stimulation (Figure 5e). Pretreatment with a Wnt antagonist (DKK1 or SFRP1), knockdown of *LRP6* transcripts using short hairpin RNA (shRNA) or E-cadherin overexpression significantly abolished Wnt/β-catenin-mediated transcriptional activation in HM20 cells. However, knockdown of *CDH1* transcripts using shRNA significantly increased transcriptional activation in LM cells (Figure 5e). To characterize the effects of Wnt signaling on promoting the CSC phenotype, we found that only low E-cadherin-expression (HM20 and LM/E-cad-shRNA) cells grew rapidly into spheres (Figure 5f) and significantly increased the SP percentage (Figure 5g) after stimulation with Wnt3a. Consistent with the results of Figure 5h, pretreatment with a Wnt antagonist (DKK1 or SFRP1), knockdown of *LRP6* transcripts or E-cadherin overexpression in HM20 cells significantly abolished the promotion of Wnt/β-catenin-elicited SP cells (Figure 5g). Equally important, the promoted cells contained a high percentage of SP, expressed at 1.5- to 2.5-fold higher levels of the ABC transporter family genes, than the control Wnt3a untreated cells (Figure 5h).

### Through endosomal sorting induced by Wnt3a treatment, the internalized E-cadherin is translocated into the nucleus, which negatively regulates Wnt/β-catenin-elicited transcriptional activity

**Figure 6:**
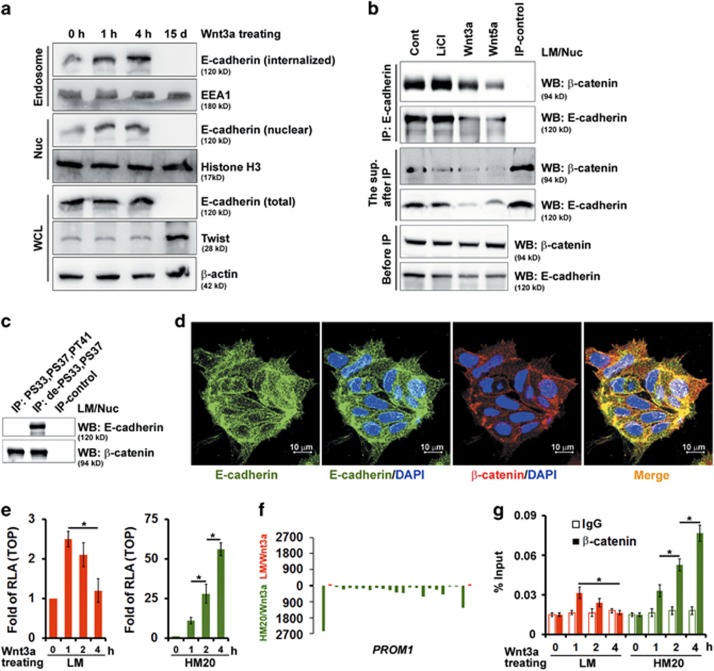
The internalized E-cadherin by endosomal sorting is translocated into the nucleus and negatively regulates Wnt/β-catenin-elicited transcriptional activity. A549 cells were cultivated in suspension for 24 days and then migrated back onto the plate to reform a monolayer (LM cells). LM cells were functionally fractionated by invasive assays, 5 times (HM5 cells), 10 times (HM10 cells) and 20 times (HM20 cells). (**a**) LM cells were treated with Wnt3a-containing medium for 1 h, 4 h and 15 days as indicated. Total cell lysates, endosomes (purified by sucrose gradient centrifugation) and nuclear (Nuc) fractions were subjected to western blotting (WB). (**b**) WB analysis of Nuc extracts of LM cells treated with control (Cont), LiCl, Wnt5a, or Wnt3a before immunoprecipitation (IP; bottom panel), in the supernatant recovered after IP with anti-E-cadherin (middle panel), and in the immunoprecipitates (top panel). (**c**) Nuc extracts were prepared from LM cells and then immunoprecipitated using antibodies against phosphorylated-S33,S37,PT41 or dephosphorylated-S33,S37 β-catenin followed by WB. (**d**) LM cells were immunostained for E-cadherin (green) and counterstained with DAPI (4',6-diamidino-2-phenylindole). Representative images taken by confocal laser microscopy are shown. Scale bars=10 μm. (**e**) TOPflash luciferase reporter assays were performed in LM (left panel) and HM (right panel) cells after treatment with control or Wnt3a-containing medium for 1, 2 and 4 h as indicated. RLA, relative luciferase activity. (**f**) ChIP sequencing (ChIP-seq) profiles for binding of β-catenin complexes in LM (red; top panel) and HM20 (green; bottom panel) cells after stimulation with Wnt3a. The illustrative region indicates *PROM1* (15,964,699–16,086,001). (**g**) ChIP–quantitative PCR analysis to validate the results of the ChIP-seq experiment described in **f**, showing the fold change of β-catenin antibody compared with the IgG control. Data in **e** and **g** were derived from three independent experiments and are presented as the mean±s.d. *P<0.05 (*t*-test).

As shown in Figure 1, IHC revealed that E-cadherin was expressed abundantly in primary lung cancers, with strong staining in the membrane (in low grade (stage IA and IB)) and the membrane/nucleus (in moderate grade (stage IIA and IIB)). To gain greater insight into the molecular mechanisms underlying tumor suppression by E-cadherin, we first examined its ability to internalize and subsequently translocate into the nucleus using the internalization assay and immunofluorescence. The kinetics of E-cadherin internalization were tracked in LM cells incubated with Wnt3a. Short-term stimulation (<4 h) of E-cadherin internalization by Wnt3a did not affect the expression level of total E-cadherin, whereas long-term stimulation (15 days) by Wnt3a significantly decreased E-cadherin expression (Figure 6a). Endocytosis of E-cadherin increased as a function of time and a greater internalization of E-cadherin was observed upon Wnt3a treatment in a time-dependent manner (short-term stimulation, <4 h). This finding suggests that Wnt signaling can accelerate its internalization (Figure 6a). Using the internalization assay, we showed that Wnt3a stimulation enhanced E-cadherin internalization and also its nuclear localization (Figure 6a). Consistent with this, increasing amounts of E-cadherin were recovered from the early endosome fraction and subsequently recovered from the nuclear fraction isolated from LM cells after Wnt3a treatment (Figure 6a). The nuclear fraction was isolated from LM cells and immunoprecipitated with a monoclonal antibody against E-cadherin. Western blotting was used to assess β-catenin and E-cadherin in the nuclear fraction before IP, in the immunoprecipitates and in the supernatant recovered after IP. Most nuclear β-catenin was associated with E-cadherin after Wnt signaling stimulation as compared with the supernatant recovered after IP, where only a very small portion of nuclear β-catenin remained (Figure 6b). In addition, this association was dependent on β-catenin activity (Figure 6c). In agreement, confocal microscopic examination revealed that endogenous E-cadherin co-localized with β-catenin and translocated into the nucleus of LM cells (Figure 6d).

Next, we tested whether nuclear E-cadherin, increased by Wnt3a-elicited endosomal sorting, exerted its transcriptional regulatory function through interaction with β-catenin. Wnt3a increased TOPflash reporter activity in HM20 cells, but not in LM cells (Figure 6e). Interestingly, Wnt3a may even decrease reporter activity in LM cells in a time-dependent manner (Figure 6e). To substantiate the results showing the different effects between LM and HM20 cells after treatment with Wnt3a, we performed chromation IP (ChIP) sequencing analysis to identify the genome-wide distribution of genes bound by the β-catenin complex in LM and HM20 cells after stimulation with Wnt3a. Figure 6f depicts a compilation of browser views of the expression of the selected gene (*PROM1*) that encode CD133 in LM (red, top panel) and HM20 (green, bottom panel) cells after stimulation with Wnt3a. We found that *PROM1* was prominently bound by β-catenin in HM20 cells whose CSC reprogramming was strongly β-catenin dependent (Figure 6f). To substantiate the results of ChIP sequencing analysis, we further performed ChIP–quantitative PCR experiments. In HM20 cells, the level of β-catenin bound to the *PROM1* proximal promoter region was significantly increased after Wnt3a stimulation, whereas it was decreased in a time-dependent manner in LM cells. These data indicated that Wnt3a induced E-cadherin internalization and nuclear translocation, which negatively regulated Wnt/β-catenin-elicited transcriptional activity in E-cadherin-proficient cells.

### A mutant E-cadherin, defective in endocytosis, inhibits its nuclear translocation and diminishes the negative effects upon Wnt/β-catenin-elicited promotion of the CSC phenotype

**Figure 7:**
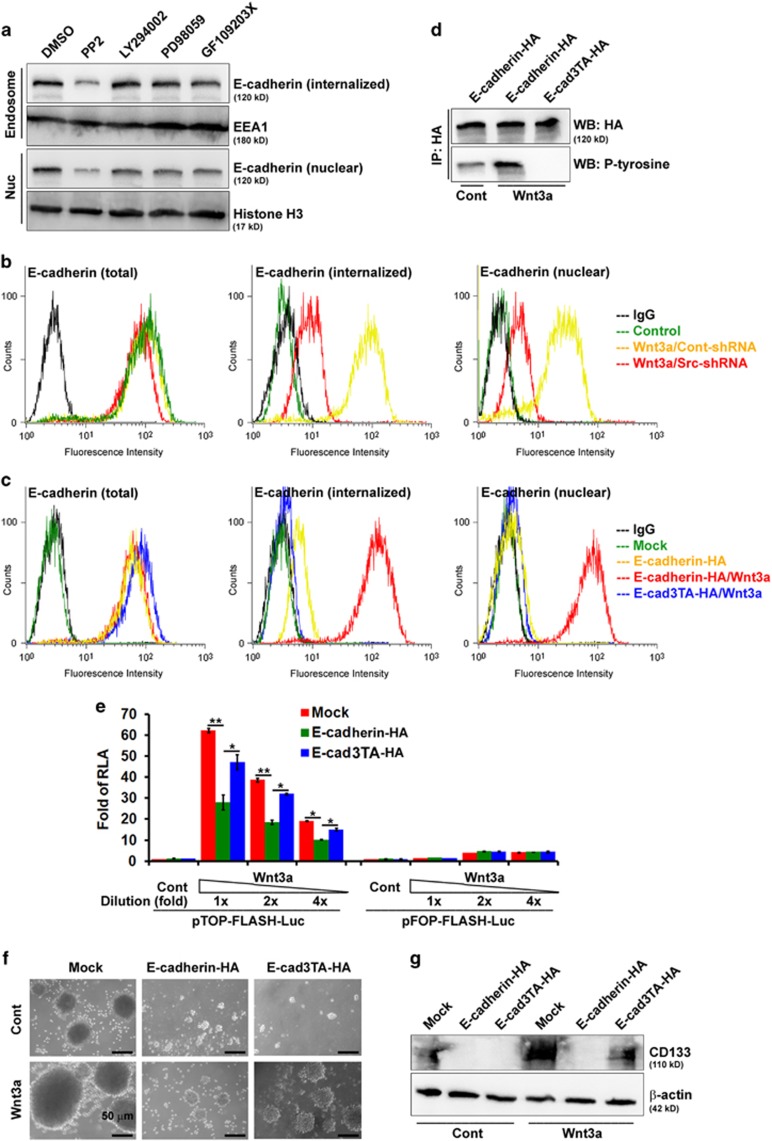
Nuclear E-cadherin is a negative regulator of Wnt/β-catenin-elicited promotion of the CSC phenotype. A549 cells were cultivated in suspension for 24 days and then migrated back onto the plate to reform a monolayer (LM cells). LM cells were functionally fractionated by invasive assays, 5 times (HM5 cells), 10 times (HM10 cells) and 20 times (HM20 cells). (**a**) Signaling pathways affecting E-cadherin endocytosis were examined by pretreating LM cells with PP2 (30 μM), LY294002 (50 μM), PD98059 (50 μM), GF109203X (5 μM) or vehicle for 30 min, followed by Wnt3a treatment. Endosomes (purified by sucrose gradient centrifugation) and nuclear (Nuc) fractions were subjected to western blotting (WB). (**b**) LM cells infected with a lentivirus encoding an shRNA targeting *SRC* before Wnt3a treatment. The details of internalization measured by flow cytometry are described in Materials and Methods. (**c**) HM20 cells transfected with plasmids encoding E-cadherin (wild-type and E-cad3TA mutant) before Wnt3a treatment. The details for internalization measured by flow cytometry are described in Materials and Methods. (**d**) Whole-cell lysates (WCLs) were prepared from HM20 cells and then immunoprecipitated using antibodies against HA or phosphorylated-tyrosine (P-Y) followed by WB. (**e**) WCLs were prepared from HM20 cells transfected with plasmids encoding wild-type and E-cad3TA before Wnt3a treatment with decreasing concentrations of Wnt3a. A wild-type (TOP)/mutant (FOP)flash luciferase reporter assay was performed. RLA, relative luciferase activity. (**f**) Microscopic analysis of spheres cultivated in suspension for 12 days; spheres were derived from HM20 cells transfected with plasmids encoding wild-type and E-cad3TA after treatment with control or Wnt3a-containing medium. (**g**) WCLs were prepared from HM20 cells transfected with plasmids encoding wild-type and E-cad3TA after treatment with control or Wnt3a-containing medium followed by western blotting (WB). Data in **e** were derived from three independent experiments and are presented as the mean±s.d. *P<0.05; **P<0.01 (*t*-test).

In epithelial cells, tyrosine kinases induce the tyrosine phosphorylation of E-cadherin, which induces endocytosis of E-cadherin.^27^ To gain more insight into the signaling pathways affecting E-cadherin endocytosis, LM cells were examined by pretreatment with pharmacologic inhibitors of specific signaling molecules or vehicles for 30 min, followed by Wnt3a treatment. As shown, treating LM cells with inhibitors selectively targeted at PI-3 kinase (LY294002), mitogen-activated protein kinase (PD98059) and protein kinase C (GF109203X) had little effect on the apparent endocytosis and subsequent nuclear translocation of E-cadherin. The blockage of those functions was accompanied by treatment with PP2, a Src kinase inhibitor (Figure 7a). We also quantified the effect of Src kinase on the internalization of E-cadherin, by using endocytosis assays to chase surface-biotinylated E-cadherin. In Src-shRNA infected LM cells, the internalization of E-cadherin was significantly decreased, compared with that of Cont-shRNA-infected cells (Figure 7b). The internalization of E-cadherin was potentiated by the addition of Wnt3a (Figures 7b and c). By contrast, E-cad3TA mutant (^753^YYY^755^→^753^AAA^755^) remained at cell–cell contacts and endocytosis was not significantly enhanced by Wnt3a (Figure 7c). Similar results were shown previously in colon cancer cell lines (Supplementary Figure 7). Tyrosine phosphorylation of wild-type E-cadherin was increased after Wnt3a treatment, whereas that of E-cad3TA mutant was abolished (Figure 7d). Taken together, these data suggest that tyrosine phosphorylation of E-cadherin by Src kinase is involved in the endocytosis of E-cadherin in epithelial cells.

To characterize the effects of internalized/nuclear E-cadherin on Wnt/β-catenin-elicited promotion of the CSC phenotype, we examined whether there were differences between wild-type and endocytosis-deficient E-cadherin in Wnt/β-catenin-elicited transcriptional activity, sphere-forming abilities and expression of CD133, a CSC marker. As shown, wild-type E-cadherin-expressing HM20 cells exhibited a two- to threefold reduction in TOPflash reporter activity as compared with mock-transfected controls. Furthermore, HM20 cells expressing E-cad3TA mutant significantly diminished the negative effect of TOPflash reporter activity as compared with wild-type E-cadherin-transfected cells (Figure 7e). These results suggested that internalized/nuclear E-cadherin also has an active role in regulating β-catenin/TCF activity. In agreement, the number and size of spheres in wild-type E-cadherin-expressing HM20 cells was dramatically decreased as compared with mock-transfected controls after stimulation with Wnt3a; however, the sphere-forming ability in E-cad3TA-expressing cells possessed little negative effect (Figure 7f). Overexpression of wild-type E-cadherin in HM20 cells abolished Wnt/β-catenin-elicited increase in CD133 expression, whereas overexpression of E-cad3TA only had a partial effect on inhibition of CD133 expression (Figure 7g). Furthermore, the blockage of TOPflash reporter activity (Supplementary Figures 8a and 9a) and Wnt/β-catenin-elicited SP cells promoting (Supplementary Figures 8b and 9b) was accompanied by treatment PP2 (a Src kinase inhibitor; Supplementary Figure 8) and overexpression of E-cad3TA mutant (endocytosis-deficient E-cadherin; Supplementary Figure 9). Taken together, internalized/nuclear E-cadherin is a more powerful negative regulator of Wnt/β-catenin-elicited promotion of the CSC phenotype.

### Mutant E-cadherin, defective in endocytosis and subsequently nuclear translocation, diminishes the negative effects upon tumor initiation in experimental animal models

For the *in-vivo* tumorigenicity assay, mice were injected subcutaneously with 10^2^–10^4^ cells. In a dose–response analysis of Mock/LM cells with or without Wnt3a treatment (10^2^–10^4^ cells injected per mouse), no tumor growth was evident at 4 weeks in the 10^2^ cell mice; 3 of 6 mice from the 10^3^ cell group had tumors and all 6 of the 10^4^ cell mice developed tumors (Table 1). LM cells transfected with E-cadherin shRNA showed a higher tumorigenic potential, with five animals developing tumors when injected with as few as 10^2^ cells (Table 1). Wnt3a stimulation promoted tumor initiation only in HM20 cells (Table 1). In addition, ectopic expression of E-cadherin in HM20 cells (10^2^ cells) significantly inhibited Wnt3a-elicited tumor initiation, whereas ectopic expression of endocytosis-deficient E-cadherin diminished effects against Wnt3a-elicited subcutaneous tumor growth (Table 1).

In this study, we found that Src kinase induced the tyrosine phosphorylation of E-cadherin, which in turn induced the endocytosis of E-cadherin and subsequent nuclear translocation; this subsequently acted as a negative regulator of Wnt signaling-elicited promotion of the CSC phenotype. β-catenin/TCF4 bound to the proximal promoter region of *PROM1*, a marker of CSCs. This association was dependent on β-catenin activity, which can be enhanced by Wnt3a stimulation. However, the β-catenin/TCF4 interaction was abolished by E-cadherin and correlated with its nuclear localization, and consequently decreased β-catenin/TCF4 transcriptional activity. Taken together, these results suggest that nuclear E-cadherin acts as a negative regulator to abolish β-catenin/TCF4 complex formation, which then inhibits binding to related gene promoters, to generate cells with properties of CSCs after Wnt3a stimulation.

## Discussion

In canonical Wnt signaling, β-catenin is the key effector responsible for transduction of the signal to the nucleus, which triggers transcription of Wnt-specific gene contribution to control the decision of cell fate. Throughout life, the level of Wnt/β-catenin-elicited transcriptional activity needs to be carefully controlled and it should neither be too high nor persist for too long. TCF/β-catenin interaction can be modulated to enhance, repress or switch off β-catenin-mediated transcription. This aspect of Wnt signaling activity and in particular transcriptional control has not yet been understood in depth. One possible way to deactivate β-catenin/TCF complex is to change the interactive partners from transcriptional activators to repressors. TCF/β-catenin transcription can be deactivated simply by separating TCF and β-catenin. Binding of β-catenin to TCF is prevented by ICAT (inhibitor of β-catenin and TCF), which interacts with the central ARM repeat of β-catenin, thereby blocking access to them.^38^ Consistent with the same idea, we also found that full-length E-cadherin can bind to the central ARM repeat of β-catenin from the cytoplasmic membrane to the nucleus, which blocks the further access of β-catenin to TCF/LEF. Therefore, full-length E-cadherin could function as an active repressor in Wnt/β-catenin-elicited transcriptional activity.

Clevers and colleagues^39^ identified two classes of β-catenin-binding sites. The first class represents the majority of the DNA-bound β-catenin and co-localizes with TCF4, the prominent TCF/LEF family member in murine intestinal epithelium, Wnt-responsive colorectal cancer cells and HEK293 embryonic kidney cells. The second class consists of β-catenin binding sites that co-localize with a minimal amount of TCF4. The latter consists of lower affinity β-catenin-binding events, does not drive transcription and often does not contain a consensus TCF-binding motif. It might suggest that membrane/E-cadherin-bound and nuclear b-catenin represent a different pool of B-catenin and is not involved in Wnt signaling.

There are several reports of cross-talk between Src and Wnt signaling. β-Catenin can be phosphorylated by Src kinase and promote its release from adhesion complexes,^40^ which has been linked to the activation of Wnt signaling.^41^ However, other studies demonstrate that tyrosine phosphorylation does not affect β-catenin abundance and/or its transcription activity.^42^ It has also been reported that Src becomes activated by Dvl2 in a Wnt-dependent manner.^43^ Recently, Davidson *et al.*^44^ proposed that LRP6 tyrosine phosphorylation by Src serves a negative regulatory function to prevent overactivation of Wnt signaling at the level of the Wnt receptor, LRP6. Src knockout cells show that a loss of LRP6 tyrosine phosphorylation is associated with a markedly increased responsiveness to Wnt3a stimulation, which suggests that Src is both necessary and sufficient for repressing Wnt/β-catenin signaling.^44^ In this study, we also found that Src kinase induced E-cadherin tyrosine phosphorylation, endocytosis and nuclear translocation, which functioned as a negative regulator of Wnt/β-catenin signaling. Taken together, Src, such as GSK3,^45^ is a multifunctional kinase in Wnt signaling and likely can function either in a positive or negative regulatory manner depending on cellular context.

Accumulating clinical evidence has demonstrated that E-cadherin,^46^ β-catenin^47^ and CD133^7^ are used as markers of malignant prognosis in different cancers, whereas their relationship has not been clearly understood. Some studies suggest that loss of E-cadherin expression does not induce Wnt signaling, whereas in other studies a role of E-cadherin in modulation of Wnt-dependent gene expression has been suggested.^48, 49^ In view of the close association between E-cadherin and β-catenin, we speculate that the nuclear localization of E-cadherin is related to β-catenin abnormalities in human lung cancer. In this study, we found that low-grade (stage IA and IB) primary lung cancers showed high levels of membranous E-cadherin and membranous β-catenin, whereas moderate-grade (stage IIA and IIB) cancers showed low-to-moderate levels of nuclear β-catenin and high-to-moderate levels of nuclear E-cadherin. In contrast, high-grade (stage III and IV) primary and metastatic lung cancers showed high levels of nuclear β-catenin and low levels of total (nuclear/membranous) E-cadherin. Loss of E-cadherin cell membrane expression may be accompanied by its detection in the nucleus, suggesting cellular redistribution during neoplasia. Furthermore, nuclear E-cadherin expression was more significantly inversely correlated with CD133 expression (*P*<0.005) than E-cadherin (total) expression (*P*<0.05), suggesting that lung cancer defined by nuclear E-cadherin^Low^/nuclear β-catenin^High^/CD133^High^ biomarkers has superior prognostic value over total E-cadherin^Low^/nuclear β-catenin^High^/CD133^High^.

## Materials and Methods

### Constructs and reagents

The E-cadherin–GFP expression construct was a gift from Alpha Yap^50^ (University of Queensland, Queensland, Australia). Wild-type E-cadherin was generated by PCR amplification of the corresponding cDNA fragments using E-cadherin–GFP as a template. The E-cadherin tyrosine-to-alanine (^753^YYY^755→753^AAA^755^) mutant was generated by site-directed mutagenesis using wild-type E-cadherin as a template. The correct clone sequence was verified by sequencing. Lentiviral-TOP-dGFP-reporter plasmids were obtained from Addgene (Addgene plasmid 14715, Cambridge, MA, USA). A pLKO.1-shRNA encoding an shRNA with a scrambled sequence or sequences targeting human Sox15 and E-cadherin, purchased from the National RNAi Core Facility, Taiwan, was introduced into HEK293T cells using the lentiviral packaging vectors pMD.G and pCMV_8.91. Antibodies against the following proteins were used: E-cadherin (610181; BD Biosciences, San Jose, CA, USA); histone H3 (Ab1791) and ABCG2 (Ab3380) (all from Abcam, Cambridge, MA, USA); active β-catenin (ALX-804-260/1; dephosphorylated at Ser33/37; Enzo Life Sciences, Farmingdale, NY, USA); total β-catenin (C2206), β-actin (A5441) and TCF4 (T5817) (all from Sigma-Aldrich, St Louis, MO, USA); CD133 (3663) and phosphorylated β-catenin (phosphorylated at Ser33/37/Thr41) (9561) (all from Cell Signaling Technology, Beverly, MA, USA).

### Human samples and IHC analysis

Sectioned human lung cancer specimens were obtained from SuperBioChips Laboratories (Seoul, South Korea). All staining procedures were performed using the Super Sensitive IHC Detection Systems kit (BioGenex, Fremont, CA, USA). Slides were dewaxed by xylene for 3 min and rehydrated in graded ethanols from 100% to 75%. Antigen retrieval was performed by boiling slides for 20 min using citrate-EDTA retrieval buffer (10 mM citrate, 2 mM EDTA, 0.05% Tween 20). Slides were blocked with 2% hydrogen peroxide for 10 min and primary antibody was used at 1:500 overnight at 4 °C. BioGenex 3,3'-diaminobenzidine and hematoxylin were used for chromogenic detection and counterstaining, respectively. A semi-quantitative method for calculating positive signals was used. Signals were counted in six fields per sample under a light microscope at × 400 magnification. The results were manually evaluated by two independent observers, to determine both the percentage of positive cells and the staining intensity as described.^35^ The observers were blind as to the stage of each sample. The IHC score was obtained by multiplying the staining intensity (0=no expression, 1=weak expression, 2=moderate expression, 3=strong expression and 4=very strong expression) by the percentage of positive cells (0=0–5% expression, 1=6–25% expression, 2=26–50% expression, 3=51–75% expression and 4=76–100% expression) in the field. The maximum possible IHC score was 4 × 4=16.

### Western blotting

Western blotting was performed as previously described.^51^

### Sphere-forming culture and the self-renewal capability assays

Spheres were generated as previously described.^52^ Briefly, cells were grown in suspension culture (1000 cells/ml) using ultra-low attachment plates (Corning, Corning, NY, USA) and serum-free RPMI (GIBCO Invitrogen, Carlsbad, CA, USA) supplemented with B27 (Invitrogen, Grand Island, NY, USA), 20 ng/ml epidermal growth factor and 10 ng/ml basic fibroblast growth factor (BD Biosciences). Spheres with a diameter of >30 μm were then counted. For serial passages (the self-renewal capability assays), spheres were collected and dissociated to single cells with trypsin and dissociated cells were re-plated in a 96-well plate (diluted to 1 cell/well in an ultra-low attachment plate) and cultured for 12 days. The spheres were then counted again. The individual spheres were found to be derived from single cells.^53^

### Identification and isolation of SP cells

SP cells were identified as previously described.^52^

### E-cadherin internalization and nuclear translocation assays

The internalization and nuclear translocation assays were performed as described.^54^ LM cells were treated with Wnt3a-containing medium for 1 h, 4 h, and 15 days as indicated. Total cell lysates, endosomes (purified by sucrose gradient centrifugation) and nuclear (Nuc) fractions were subjected to western blotting. For internalization measured by flow cytometry, cells were incubated with sulfo-NHS-SS-biotin-conjugated anti-E-cadherin mAb at 4 °C for 1 h followed by further incubation with Wnt3a at 37 °C for 2 h. Cells were incubated with 0.1 M glycine in phosphate-buffered saline for 30 min at 4 °C, to quench the unreacted biotin (for total E-cadherin detection). Surface-retained biotin was removed using reduced glutathione (50 mM glutathione, 75 mM NaCl, 1 mM EDTA, 1% bovine serum albumin and 0.75% (vol/vol) of 10 N NaOH, for internalized E-cadherin detection). After cytoplasm membrane lysis, nuclei were isolated (for nuclear E-cadherin detection). Cells and nuclei were fixed and stained by avidin-Alexa 488 for flow cytometry analysis. The expression level of E-cadherin was calculated by setting the fluorescence intensity of cells after biotin labeling but without glutathione incubation as ‘total'. The expression level of internalized E-cadherin was calculated by setting the fluorescence intensity of cells after biotin labeling followed by glutathione incubation as ‘internalized'. The expression level of nuclear E-cadherin was calculated by setting the fluorescence intensity of cells after biotin labeling, glutathione incubation and cytoplasm membrane lysis and nuclei isolation as ‘nuclear'.

### ChIP–quantitative PCR

ChIP assays were performed as previously described.^54^

### Animal experiments

The murine studies were conducted in accordance with the Institutional Animal Care and Use Committee guidelines and were approved by the Animal Care and Use Committee of National Tsing Hua University. Severe combined immunodeficient CB17 female mice (6 weeks old) were used. For the *in-vivo* tumorigenicity assay, mice were injected subcutaneously with 10^2^–10^4^ cells in 100 μl of a 1:1 mixture of Dulbecco's modified Eagle's medium (with or without Wnt3a)/Matrigel. Tumorigenicity was evaluated at 4 weeks after transplantation.

### Statistical analysis

Statistical analysis of data was performed by Student's *t*-test using Microsoft Excel (Microsoft Office Professional Plus 2013). Spearman's rank correlation coefficient was used to assess the association between two continuous variables. Differences were considered to be statistically significant at *P*<0.05.

## Figures and Tables

**Table 1:** *In-vivo* tumorigenicity assay

	*Tumorigenesis (SC*)		
	*10*^*4*^ *cells*	*10*^*3*^ *cells*	*10*^*2*^ *cells*
*LM*			
Cont			
Mock	6/6	3/6	0/6
Wnt3a			
Mock	6/6	3/6	0/6
E-cad-shRNA		6/6	5/6

*HM20*			
Cont			
Mock		6/6	3/6
Wnt3a			
Mock		6/6	6/6
E-cadherin-HA		4/6	0/6
E-cad3TA-HA		5/6	3/6

Abbreviation: SC, subcutaneous.

For *in-vivo* tumorigenicity assay, mice were injected subcutaneously with 10^2^–10^4^ cells. Tumorigenecity was evaluated at 4 weeks after transplantation
