# Supplementary figures and images for: An aberrant nuclear localization of E-cadherin is a potent inhibitor of Wnt/β-catenin-elicited promotion of the cancer stem cell phenotype
Source: Oncogenesis. 2015 Jun 15;4(6):e157–. doi: 10.1038/oncsis.2015.17 (PMC4491612; doi:10.1038/oncsis.2015.17)

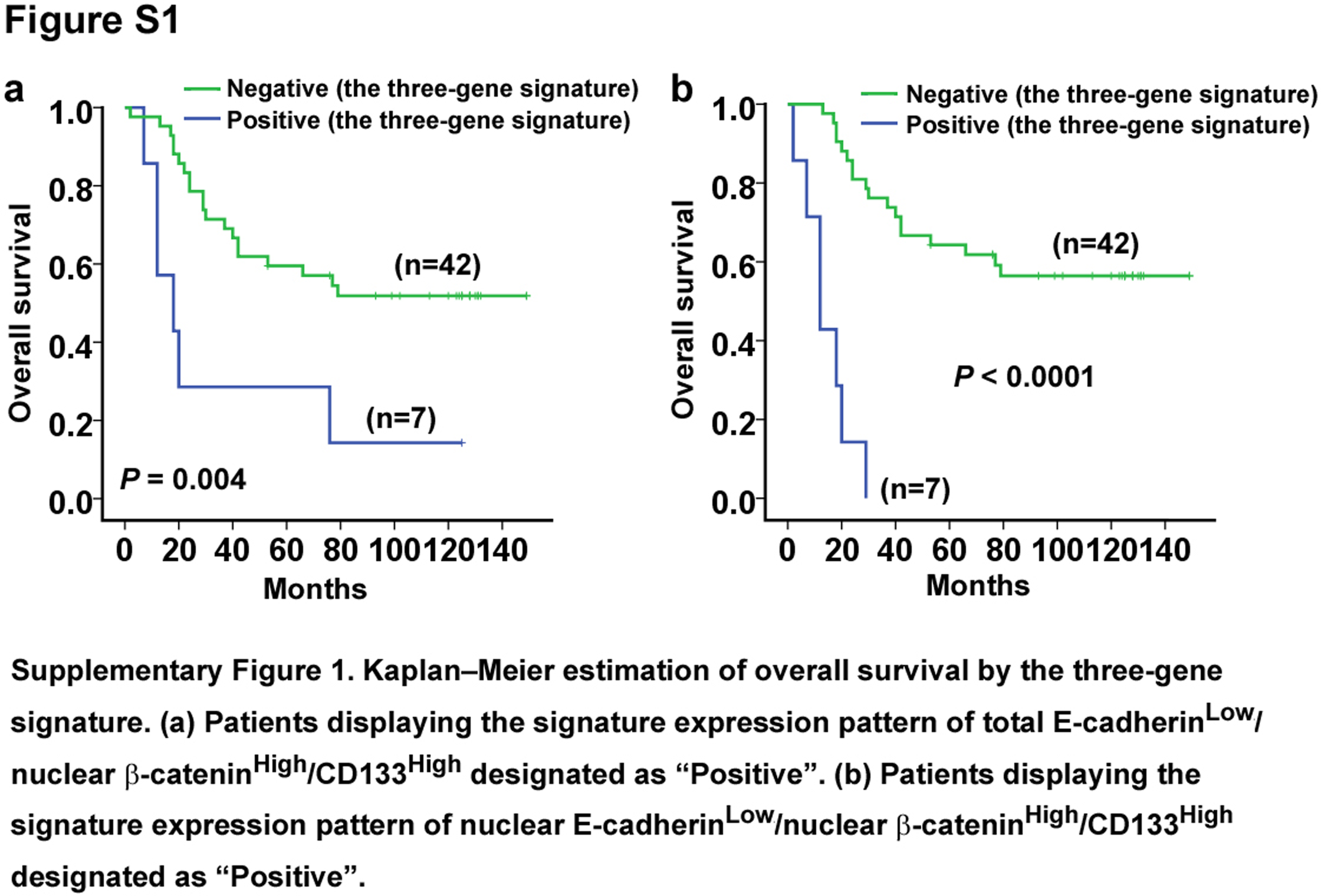

Supplement: Supplementary Figure 1 [file oncsis201517x1.tif]

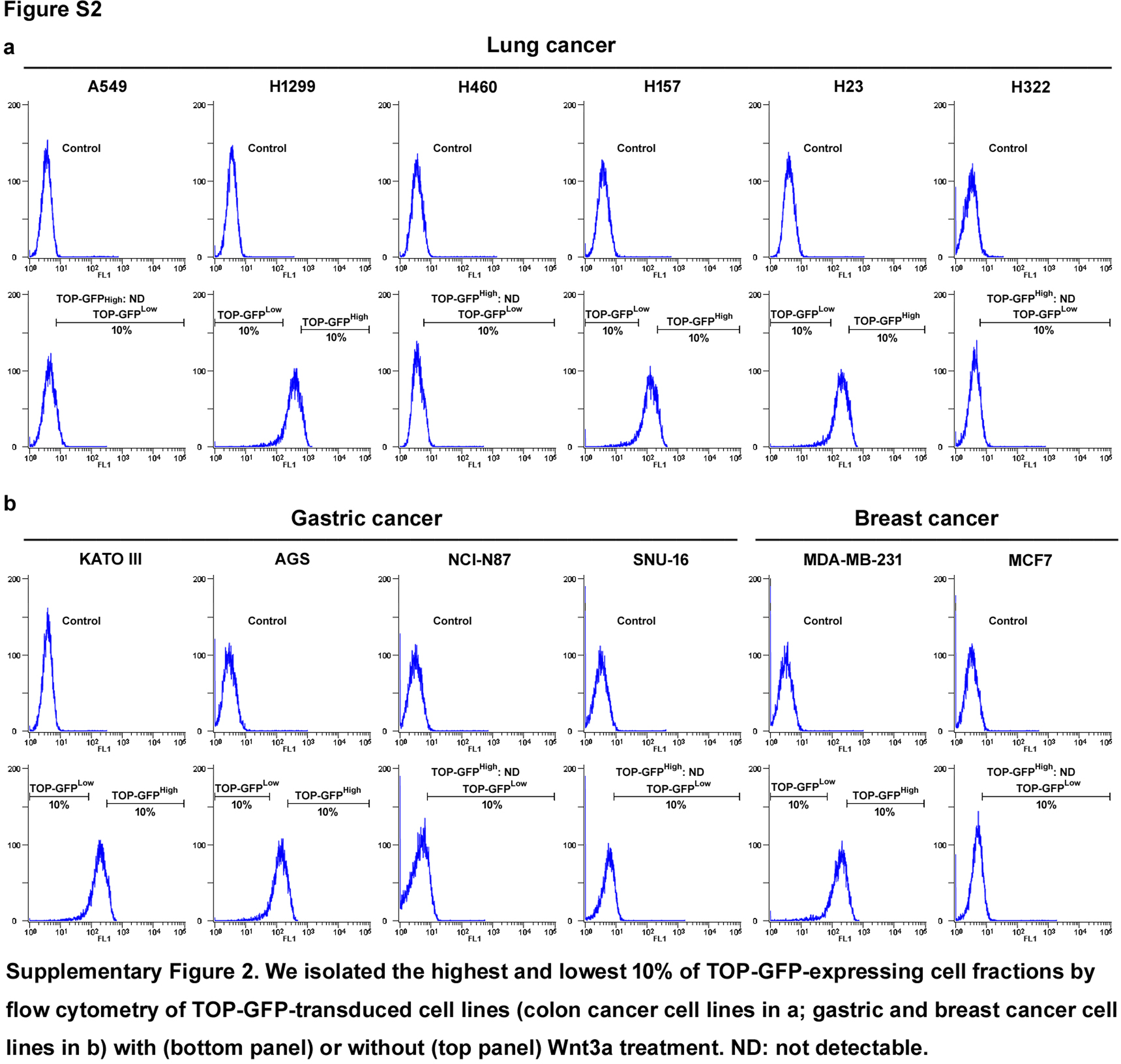

Supplement: Supplementary Figure 2 [file oncsis201517x2.tif]

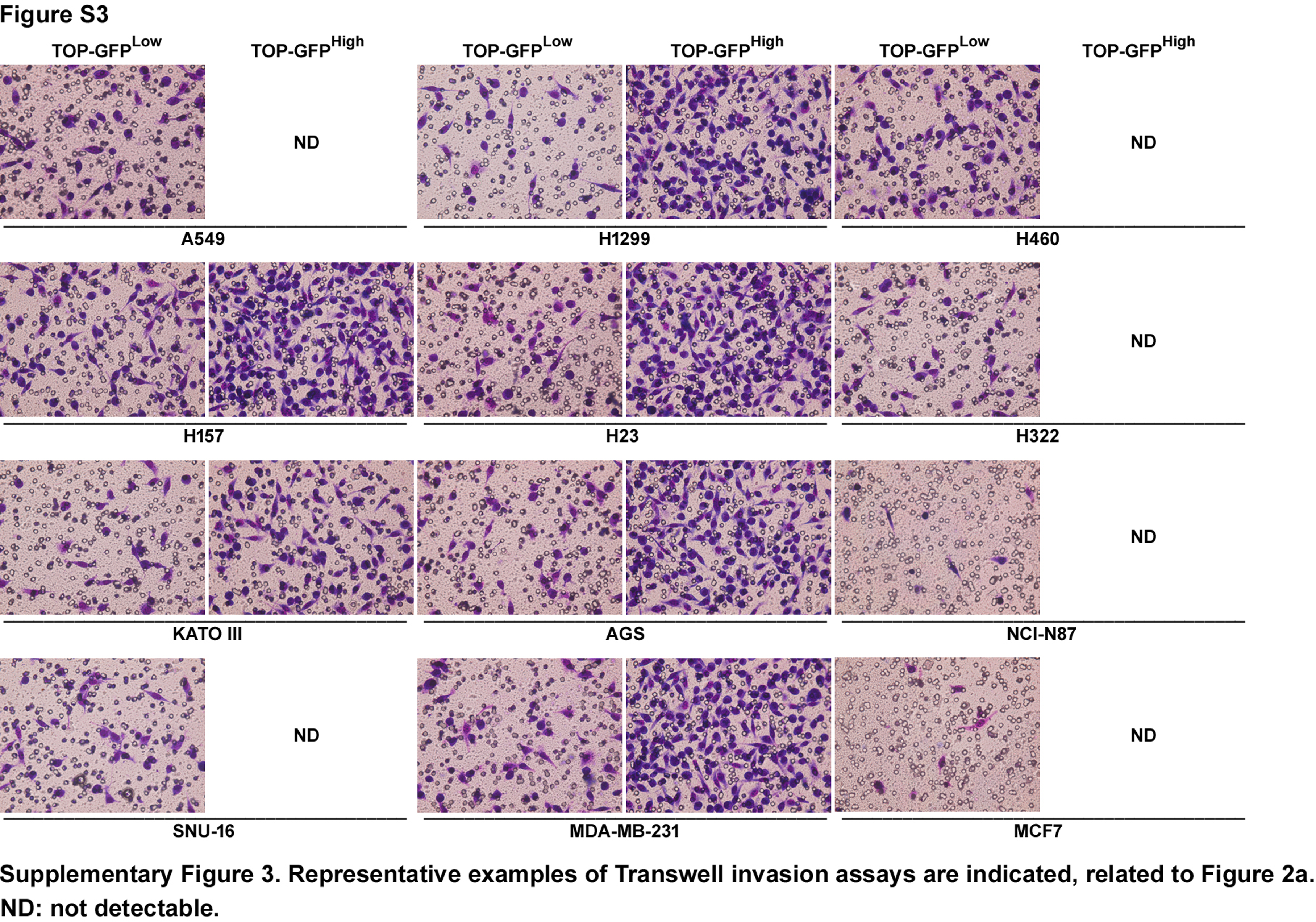

Supplement: Supplementary Figure 3 [file oncsis201517x3.tif]

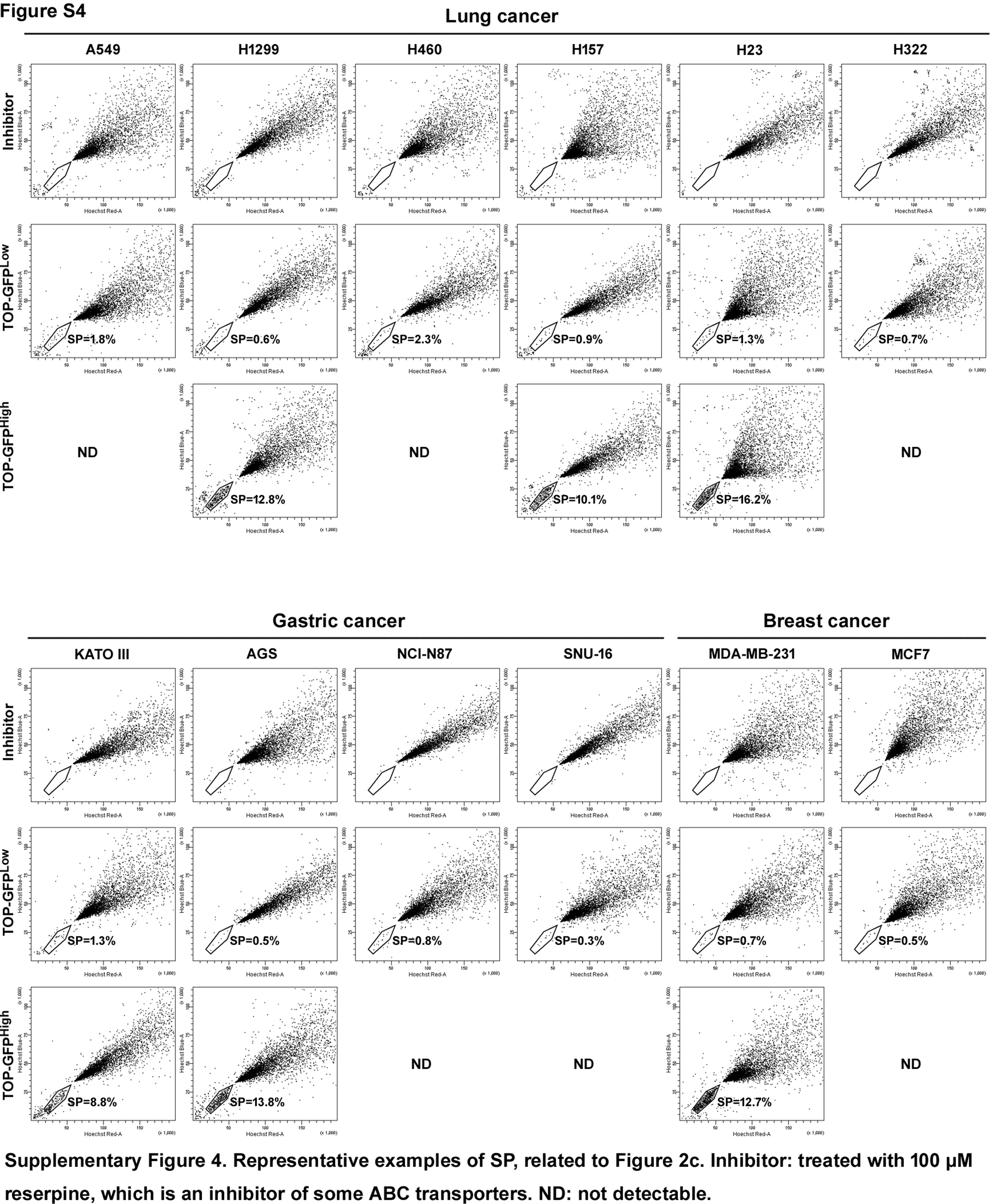

Supplement: Supplementary Figure 4 [file oncsis201517x4.tif]

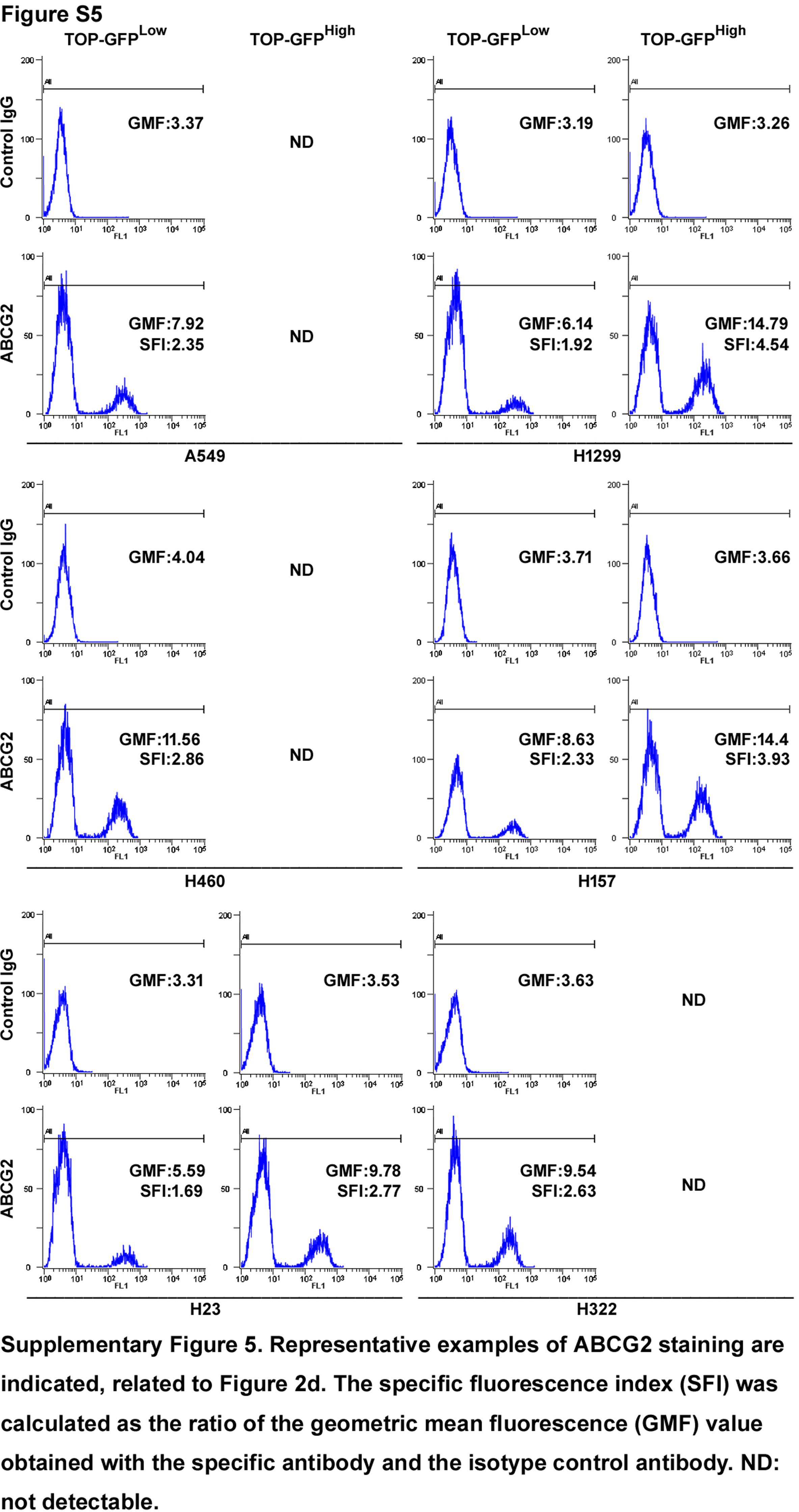

Supplement: Supplementary Figure 5 [file oncsis201517x5.tif]

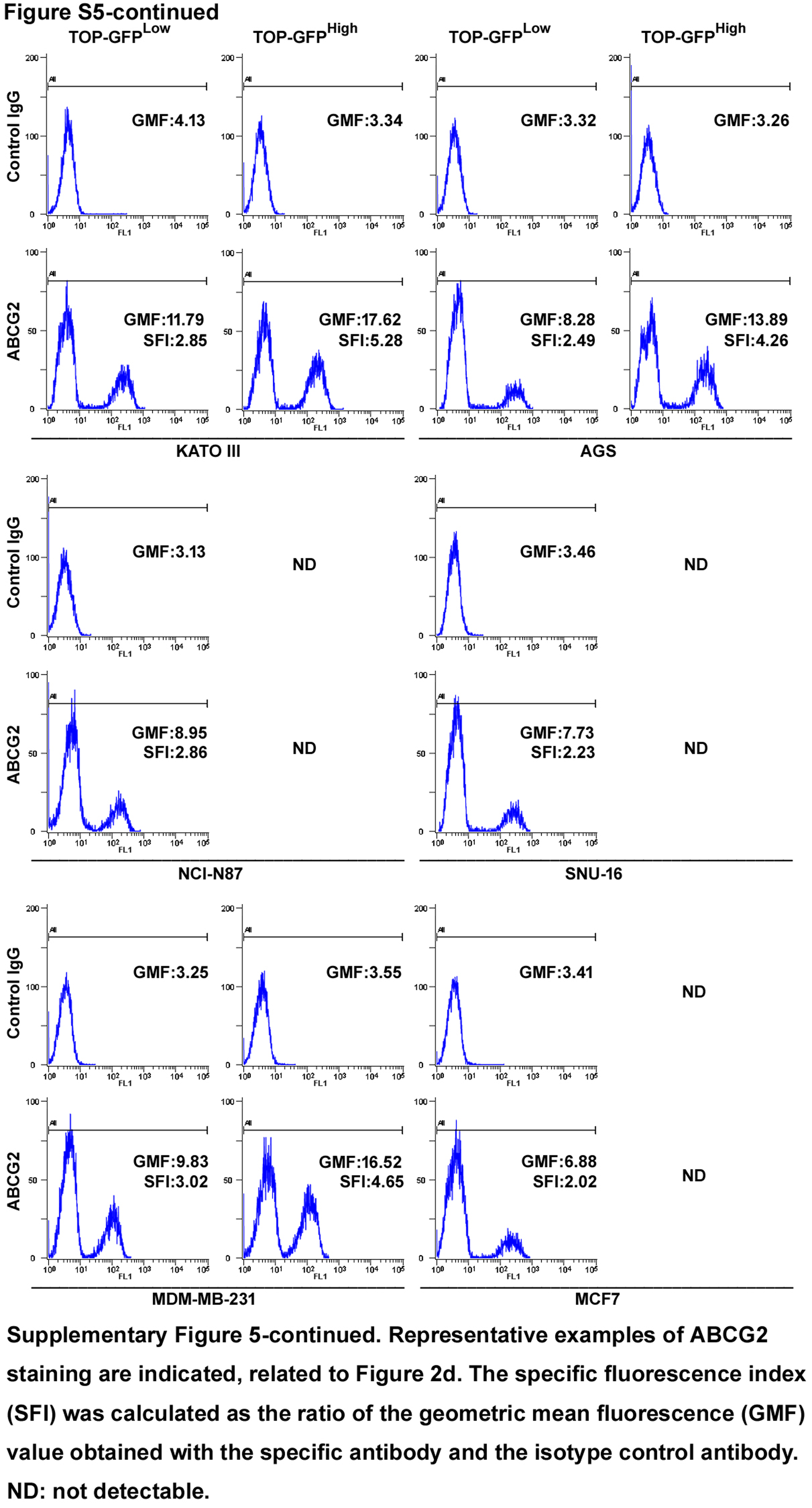

Supplement: Supplementary Figure 5 [file oncsis201517x6.tif]

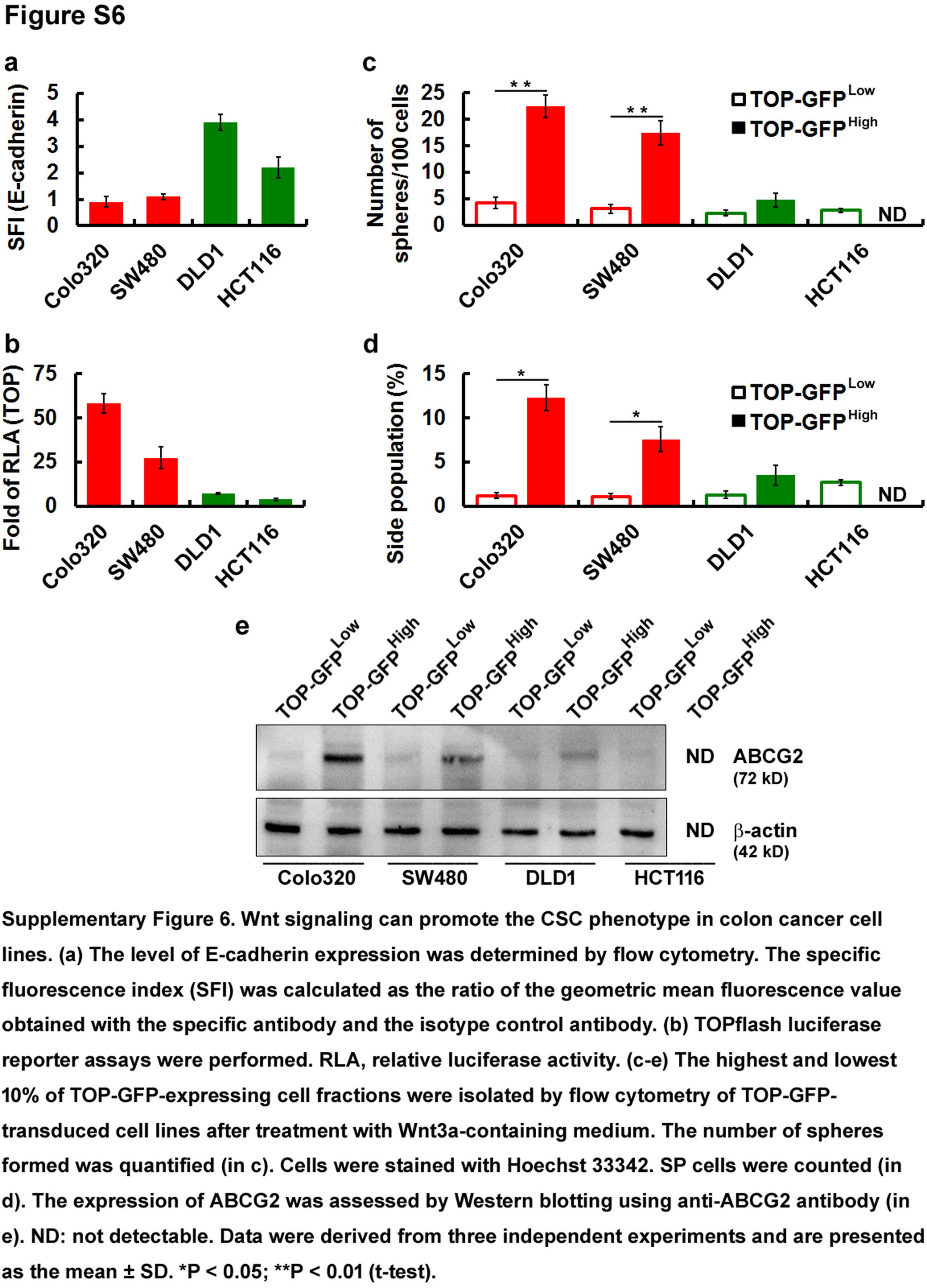

Supplement: Supplementary Figure 6 [file oncsis201517x7.tif]

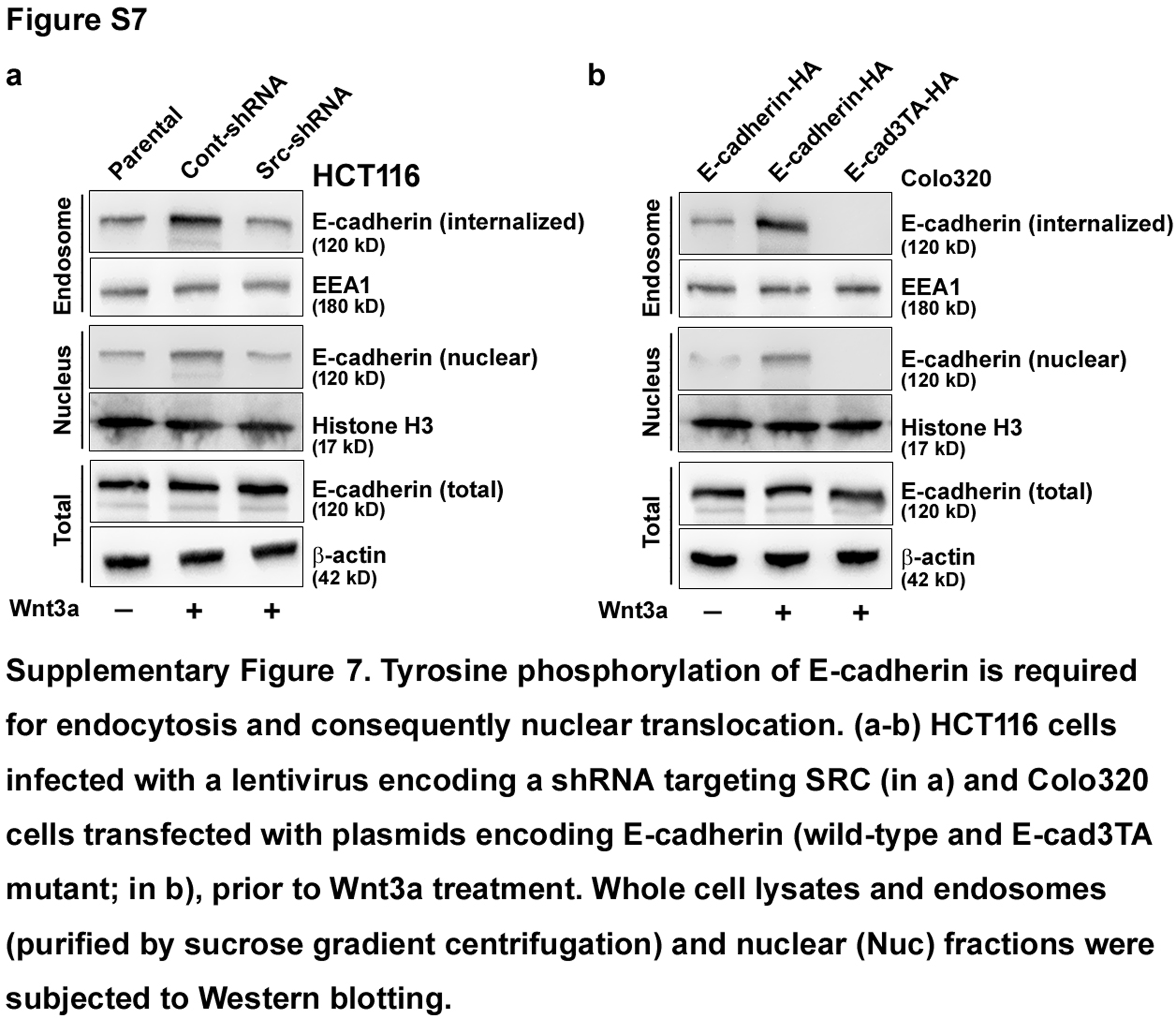

Supplement: Supplementary Figure 7 [file oncsis201517x8.tif]

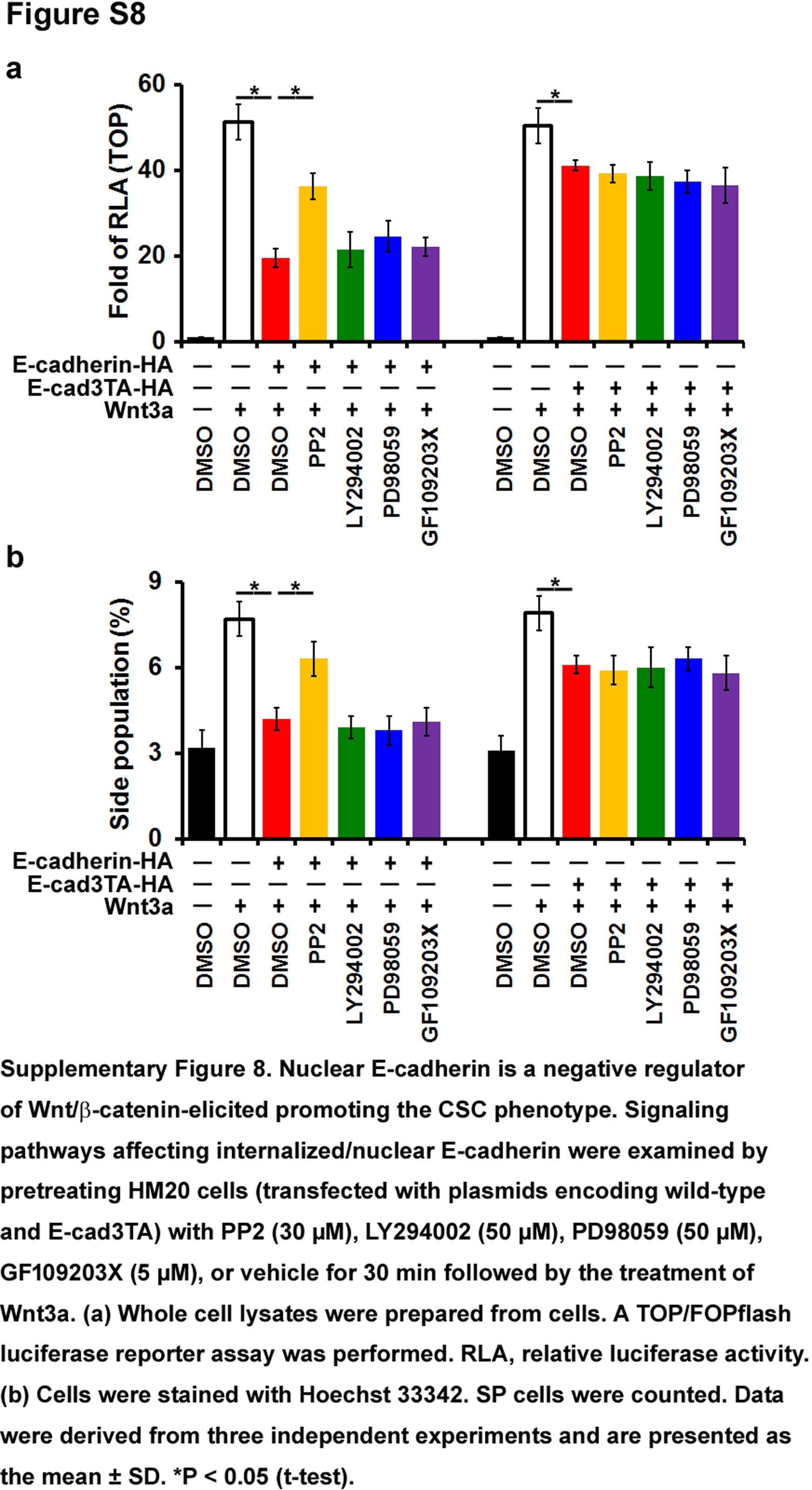

Supplement: Supplementary Figure 8 [file oncsis201517x9.tif]

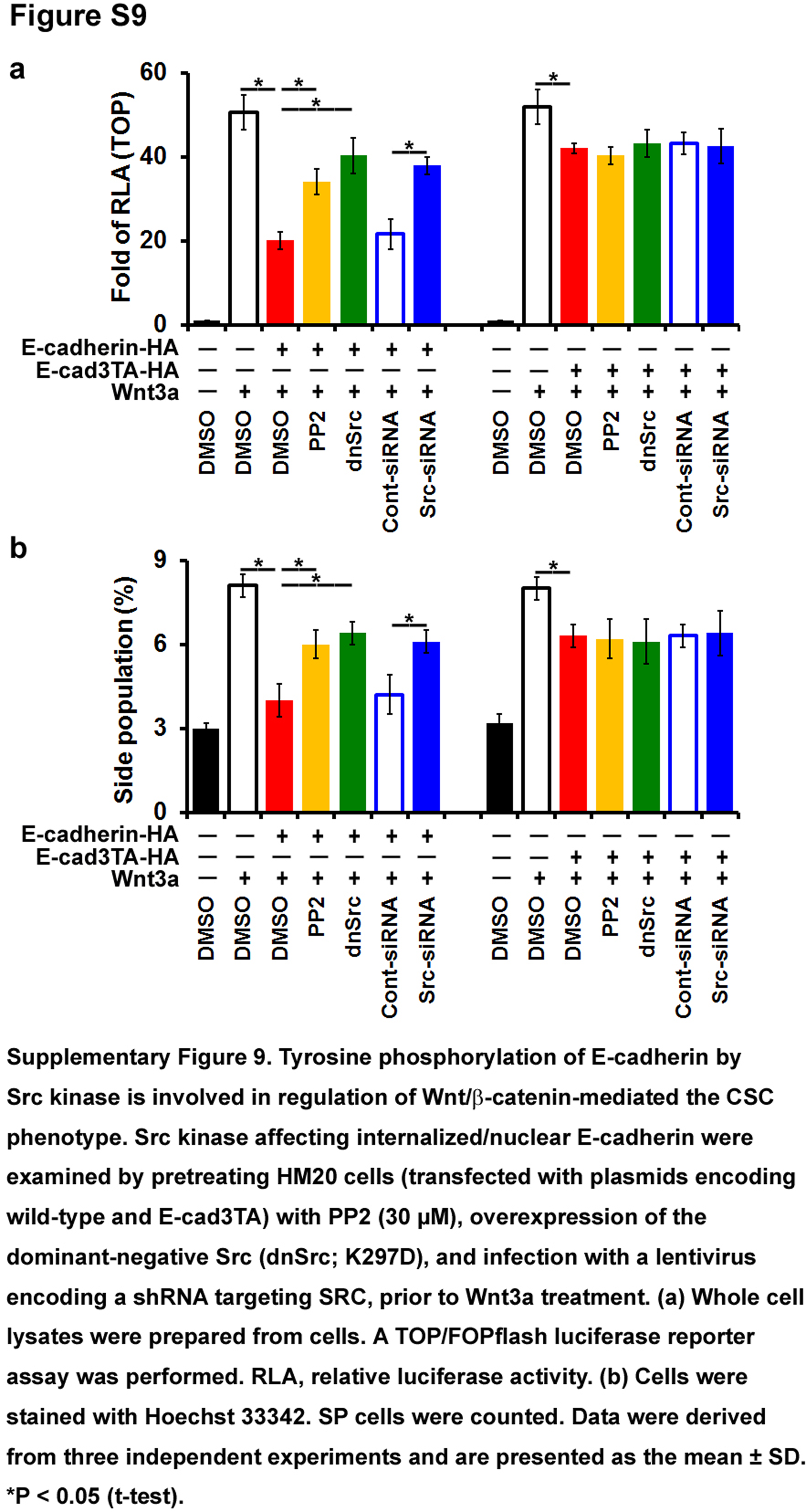

Supplement: Supplementary Figure 9 [file oncsis201517x10.tif]
